# Adsorption and Detection of Toxic Gases on CuO-Modified SnS Monolayers: A DFT Study

**DOI:** 10.3390/s25051439

**Published:** 2025-02-26

**Authors:** Xinyue Liang, Ping Wang, Kai Zheng, Xuan Yang, Meidan Luo, Jiaying Wang, Yujuan He, Jiabing Yu, Xianping Chen

**Affiliations:** 1 The State Key Laboratory of Power Transmission Equipment & System Security and New Technology, Chongqing University, Chongqing 400044, Chinaxianpingchen@cqu.edu.cn (X.C.); 2 Key Laboratory of Optoelectronic Technology & Systems, Education Ministry of China, College of Optoelectronic Engineering, Chongqing University, Chongqing 400044, Chinayujiab@cqu.edu.cn (J.Y.)

**Keywords:** CuO-SnS monolayer, first-principles calculations, gas sensors, toxic gas detection

## Abstract

The emission of toxic gases such as NO_2_, NO, SO_2_, and CO from industrial activities, transportation, and energy production poses significant threats to the environment and public health. Traditional gas sensors often lack high sensitivity and selectivity. To address this, our study uses first-principles density functional theory (DFT) to investigate CuO-SnS monolayers for improved gas sensor performance. The results show that CuO modification significantly enhances the adsorption capacity and selectivity of SnS monolayers for NO_2_ and NO, with adsorption energies of −2.301 eV and −2.142 eV, respectively. Furthermore, CuO modification is insensitive to CO_2_ adsorption, demonstrating excellent selectivity. Structural and electronic analyses reveal that CuO modification reduces the band gap of SnS monolayers from 1.465 eV to 0.635 eV, improving the electrical conductivity and electron transfer, thereby enhancing the gas adsorption sensitivity. Further analyses highlight significant electronic interactions and charge transfer mechanisms between CuO-SnS monolayers and NO_2_ and SO_2_ molecules, indicating strong orbital hybridization. In conclusion, this study provides a theoretical basis for developing high-performance gas sensors, showing that CuO-SnS monolayers have great potential for detecting toxic gases.

## 1. Introduction

NO_X_, SO_2_, and CO are prevalent atmospheric pollutants, primarily originating from industrial activities, transportation, and energy production [1,2]. NO_X_ is a major cause of acid rain, which can damage ecosystems, erode buildings, and negatively impact soil and water quality [3,4]. SO_2_, a byproduct of burning fossil fuels such as coal and petroleum-based fuels, is also a typical emission from industrial processes [5]. CO mainly arises from combustion processes, including vehicle emissions, industrial production, and heating devices [6]. The emission of these gases not only contaminates the natural environment but also presents substantial health hazards, including respiratory infections, decreased lung function, and neurological damage [7]. Therefore, the dependable detection and absorption of these toxic gases are of utmost significance.

Compared to traditional gas sensors, two-dimensional (2D) materials have drawn substantial attention due to their high specific surface area and nanoscale layered structures, along with their outstanding chemical activity and selectivity [8,9,10,11]. Doping 2D materials with noble metals such as Pd, Pt, Au, and Ag is a common strategy to enhance gas response sensitivity, selectivity, and response–recovery times [12,13,14]. Nevertheless, the intense surface activity of metal atoms can lead to oxidation during prolonged operation, significantly reducing sensor stability. To address this issue, metal oxides such as CuO, Ag_2_O, and ZnO are widely employed to modify 2D materials, improving their chemical stability and surface activity [15,16,17,18,19]. For example, Guo et al. synthesized Ti_3_C_2_T_X_/CuO nanocomposites via a hydrothermal method, showing that a NO₂ sensor outperformed pure Ti_3_C_2_T_X_ in response speed, selectivity, and stability at room temperature [20]. Huang et al. developed an rGO/PPy/Cu_2_O-based gas sensor with high sensitivity, selectivity, and repeatability for NO_2_ detection [21]. Haibo Dong et al. demonstrated that Ag_2_O-doped InN enhances gas sensing for SOF_4_, SOF_2_, and SO_2_F_2_ while limiting NO_2_ detection, offering a basis for GIS partial discharge monitoring [22].

Monolayer SnS is a novel 2D material with excellent thermoelectric properties, making it suitable for applications in biomedicine, memristors, sensors, and the battery industry [23,24,25]. Several studies have demonstrated the feasibility of SnS in gas detection. Luyen et al. developed Ag/SnS composites on SnS films, which showed high carrier mobility and exceptional sensitivity for detecting NO_2_ gas [26]. Qin et al. investigated the impact of gas adsorption and vacancy defects on the properties of SnS materials through DFT calculations [27]. Additionally, Guo et al. conducted DFT-based research, revealing that Ag-doped monolayer SnS exhibits significant adsorption capabilities for NO₂ and NO gas molecules, indicating its potential for monitoring toxic gases [28].

In this work, copper oxide (CuO) nanoclusters were chosen to modify SnS to investigate its adsorption behavior for NO_2_, NO, CO_2_, CO, and SO_2_ based on DFT. The adsorption properties of these gas molecules on the surface of the CuO-SnS monolayers were further understood by analyzing the adsorption properties such as the geometrical structure of the adsorption system, the energy bands, the adsorption energy, and the charge transfer. In order to deeply explore the mechanism of the adsorption reaction, the density of states (DOS), differential density charge (CDD), and electron localization function (ELF) were also combined for detailed analysis. Finally, we also calculated the adsorption recovery time for each gas molecule and evaluated the regeneration capacity and lifetime of the CuO-SnS monolayers in practical applications. These findings offer theoretical guidance for the design and development of efficient gas sensing materials and adsorbents.

## 2. Computational Details

We utilized the Dmol^3^ module within the Materials Studio software (version 2020) based on DFT calculations [29]. The calculations employed the Perdew–Burke–Ernzerhof (PBE) generalized gradient approximation (GGA) for the exchange–correlation functional [30,31,32]. Grimme dispersion correction accounted for the effect of weak interactions and ensured computational accuracy [33]. The surface adsorption behavior of gases on SnS monolayers before and after the introduction of metal oxide doping was thoroughly analyzed. Considering the limitation of computational resources, we constructed a supercell monolayer with dimensions of 3 × 3 × 1, containing 18 Sn atoms and 18 S atoms [34,35,36]. To avoid the effect of interactions between neighboring layers, the vacuum layer was increased to 25 Å in the z-direction [37].

We employed the Direct Inversion in the Iterative Subspace (DIIS) method to enhance the convergence speed of the self-consistent field (SCF) calculations [38]. The total energy convergence tolerance for all geometry optimizations was established at 1 × 10^−5^ Ha, the maximum force at 2 × 10^−3^ Ha/Å, the maximum displacement at 5 × 10^−3^ Å, and the SCF convergence accuracy at 1.0 × 10^−6^ Ha [39]. The Brillouin zone sampling densities for geometry optimization and electronic property calculations were set to 5 × 5 × 1 and 7 × 7 × 1, respectively [40,41]. In addition, the COSMO solvation model was used to simulate the effect of humidity in the actual environment.

The energy released (*E*_b_) during the binding process of CuO with the SnS surface is defined by following equation:

(1) $$E_{b}=E_{CuO\text{-}SnS}-E_{SnS}-E_{CuO}$$

where *E*_CuO-SnS_, *E*_SnS_, and *E*_CuO_ represent the CuO-SnS monolayer, pure SnS monolayer, and CuO unit, respectively. When *E*_b_ is negative, it indicates that the binding process between CuO and SnS is accompanied by a net energy release, and the system spontaneously evolves toward a stable state, following the second law of thermodynamics. The larger the absolute value of *E*_b_, the stronger the chemical bonding interaction at the CuO-SnS interface, leading to a more stable structure of the composite system.

The adsorption energy (*E*_ads_) is calculated using Definition (2):

(2) $$E_{ads}=E_{Gas/CuO\text{-}SnS}-E_{CuO\text{-}SnS}-E_{Gas}$$

where *E*_Gas/CuO-SnS_, *E*_CuO-SnS_, and *E*_Gas_ represent the energy of the doped system following gas adsorption, the energy of the standalone substrate system, and the energy of the individual gas, respectively. If *E*_ads_ is negative, it indicates energy release during the reaction process, making the process spontaneous.

The high-precision Hirshfeld analysis method was employed to calculate the charge transfer amount (*Q*) between gas molecules and the CuO-SnS monolayer, as determined by Equation (3):

(3) $$Q=Q_{ads}-Q_{iso}$$

where *Q*_ads_ represents the gas’s net charge following adsorption, and *Q*_iso_ represents the net charge of the gas prior to adsorption.

The band gap (*E*_g_) is defined by Equation (4):

(4) $$E_{g}=|E_{CBM}-E_{VBM}|$$

where *E*_CBM_ represents the energy of the lowest conduction band, while *E*_VBM_ denotes the energy of the highest valence band [42].

## 3. Results and Discussion

### 3.1. Optimized Structures of Gases, Pure SnS Monolayer, and CuO-Modified SnS Monolayer

**Figure 1:**
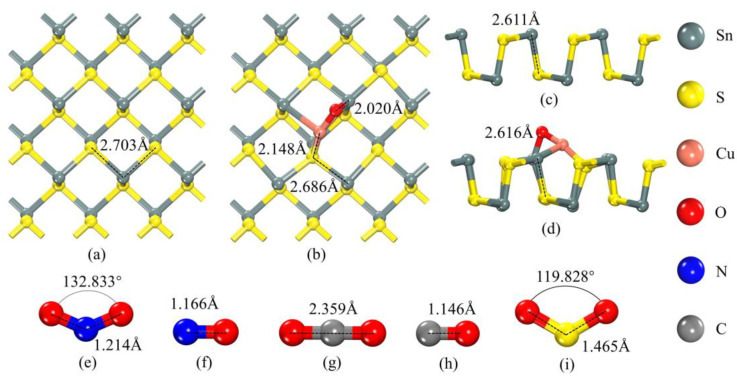
Geometric structures of pure SnS and CuO-SnS: (**a**–**d**) top and side views. Geometric structures of (**e**) NO_2_, (**f**) NO, (**g**) CO_2_, (**h**) CO, and (**i**) SO_2_ molecules.

The optimized monolayer SnS structure is illustrated in Figure 1a,c, showing that each Sn atom is connected to S atoms through covalent bonds, forming a continuous zigzag pattern. The Sn-S bond lengths measure 2.703 Å and 2.611 Å, respectively. To achieve the most stable CuO-SnS structure, we explored different modifying sites. Due to space limitations, details of the models and numerical comparisons are provided in the Supplementary Materials. By evaluating the highest binding energy (*E*_b_) of −3.572 eV, the most stable configuration is shown in Figure 1b,d. Following the addition of CuO, noticeable alterations in the structure of SnS are apparent, with the internal rhombic lattice becoming asymmetric and displaying different bond lengths. This modification process suggests the presence of a strong chemical interaction. The Cu atom bonds with the S atom in the SnS monolayer, forming a Cu–S bond with a length of 2.148 Å. Similarly, the O atom bonds with the Sn atom in the SnS monolayer, forming an O–Sn bond with a length of 2.020 Å. These interactions further modify the original structure of the SnS monolayer.

Figure 1e–i display the optimized geometries of various gas molecules, along with their bond lengths and bond angles. Figure 1e shows the NO_2_ molecule with an O-N-O bond angle of 132.833° and a N-O bond length of 1.214 Å. Figure 1f depicts the NO molecule with a N-O bond length of 1.166 Å. Figure 1g illustrates the CO_2_ molecule with an O-C-O bond length of 2.359 Å. Figure 1h presents the CO molecule with a C-O bond length of 1.146 Å. Finally, Figure 1i displays the SO_2_ molecule, where the O-S-O bond angle is 119.828° and the S-O bond length measures 1.465 Å. These structures highlight the optimized geometries of different molecules, providing a foundation for further investigation of their electronic properties and interactions.

**Figure 2:**
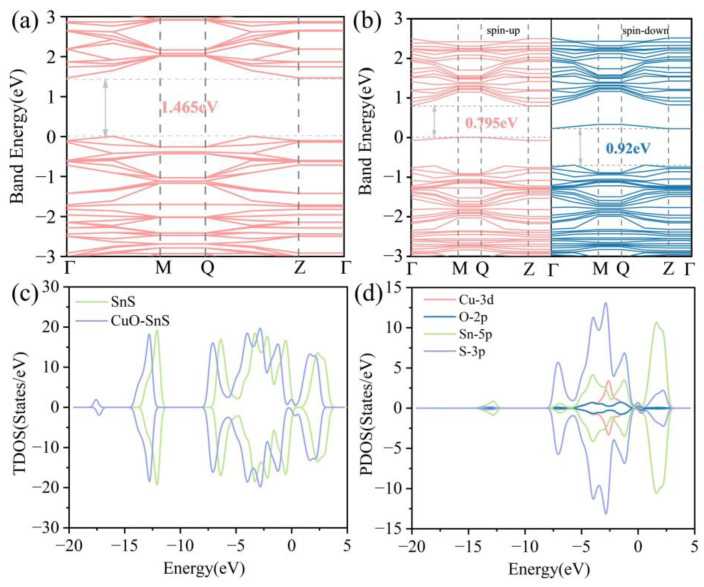
The band structures of (**a**) pure SnS and (**b**) CuO-SnS. The TDOS of (**c**) CuO-SnS and PDOS of (**d**) CuO-SnS.

The modification with CuO significantly alters the electronic structure of SnS. By analyzing the band structure and density of states (DOS), the electronic properties of the modified material can be predicted. In Figure 2a, the band structure of the pure SnS monolayer shows a band gap of 1.465 eV, indicating that it is a semiconductor with low electrical conductivity in the absence of external energy input. However, after CuO modification, the system exhibits spin-polarized band gaps of 0.795 eV for the spin-up channel and 0.92 eV for the spin-down channel, as illustrated in Figure 2b, highlighting the effect of magnetic splitting induced by the unpaired electrons of CuO. This spin-asymmetric band gap modulation significantly enhances the electronic states near the Fermi level, facilitating orbital hybridization with adsorbed gas molecules and improving charge transfer efficiency. Furthermore, the reduced band gap lowers the energy barrier for electron transport, thereby optimizing the conductivity response upon gas adsorption.

From the total density of states (TDOS) in Figure 2c, it can be seen that the DOS near the Fermi level significantly increases after CuO modification, consistent with the reduced band gap observed in the band structure. This increase is observed for both the spin-up and spin-down states. Figure 2d presents the partial density of states (PDOS) for different atoms, where the introduction of Cu-3d and O-2p states contributes additional electronic states near the Fermi level. This is crucial for enhancing the material’s electron transport properties.

### 3.2. Gas Adsorption Analysis on SnS and CuO-SnS Monolayer Surface

#### 3.2.1. Structure Analysis

**Figure 3:**
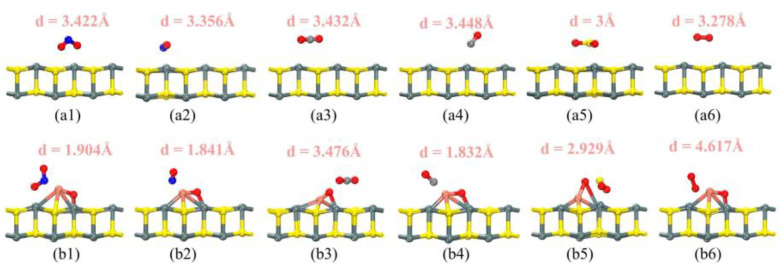
The most stable structure of the system after gas adsorption: (**a1**) NO_2_, (**a2**) NO, (**a3**) CO_2_, (**a4**) CO, (**a5**) SO_2_, and (**a6**) O_2_ adsorbed on pure SnS; (**b1**) NO_2_, (**b2**) NO, (**b3**) CO_2_, (**b4**) CO, (**b5**) SO_2_, and (**b6**) O_2_ adsorbed on CuO-SnS.

During this investigation, we explored various adsorption sites by positioning different atoms of the gas molecules above the corresponding Cu and O atoms of the substrate. Simultaneously, adsorption models for pristine SnS were constructed, with the gas atoms corresponding to the S and Sn atoms of the substrate, respectively, in order to cover as many possible models as possible. Due to space limitations, the detailed numerical analysis is provided in the Supplementary Materials. The stability of the adsorption system was initially evaluated based on the magnitude of the adsorption energy, with larger absolute values indicating greater stability. Figure 3(a1,a2,b1,b2) display the most stable optimized structures of SnS and CuO-SnS monolayers after the adsorption of gases (NO₂, NO, CO₂, CO, SO₂, O₂). The optimal adsorption sites are clearly identifiable in these figures. Following gas adsorption, both the substrate and the gas molecules exhibit varying degrees of deformation. Our study primarily focuses on the electronic structural aspect; hence, we will not analyze the specifics in this context.

**Figure 4:**
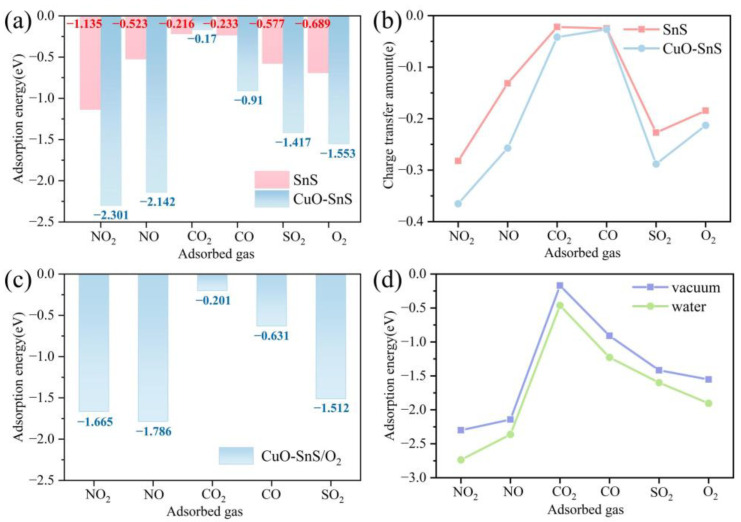
Gas adsorption on pure SnS and CuO-SnS surfaces: (**a**) Adsorption energies of gases on SnS and CuO-SnS. (**b**) Electron transfer upon gas adsorption on SnS and CuO-SnS. (**c**) Adsorption energies of gases in the presence of O_2_. (**d**) Adsorption energies of gases on CuO-SnS in different environments.

As illustrated in Figure 4a, CuO modification substantially improved the adsorption energies of most gases on SnS, except for CO_2_. Specifically, the adsorption energies of NO_2_ and NO increased to −2.3 eV and −2.142 eV, respectively, which were approximately 2-fold and 4-fold higher than those of pristine SnS. This enhancement is attributed to the reconstructed surface electronic structure induced by CuO incorporation. Further analysis of electron transfer, shown in Figure 4b, revealed a notable increase in charge redistribution during gas adsorption, particularly for NO_2_ and NO, confirming that CuO doping effectively optimizes the gas adsorption performance of SnS by strengthening interactions at active sites. Under simulated practical conditions, the introduction of O_2_ resulted in a reduction in adsorption energies for all gases on CuO-SnS, as shown in Figure 4c. However, key characteristics such as the selectivity and adsorption energy remained stable. Additionally, humidity significantly enhanced the adsorption energies in aqueous environments, as depicted in Figure 4d, which indicates the promoting effect of humidity on the adsorption kinetics of the CuO-SnS surface.

Subsequently, the adsorption performance parameters of CuO-SnS for six gases were analyzed, with the corresponding values presented in Table 1. The data indicate that the adsorption energies follow the trend NO_2_ > NO > O_2_ > SO_2_ > CO > CO_2_. The strongest adsorption capacities of the CuO-SnS monolayer are observed for NO_2_ and NO, with adsorption energies of −2.301 eV and −2.142 eV, respectively. Typically, when the energy level is above −0.6 eV, it indicates physical adsorption. So, we have reason to speculate that a chemical reaction has occurred. The adsorption energy of SO_2_ is −1.414 eV, which also suggests a strong interaction. CO exhibits a moderate adsorption energy of −0.910 eV, indicating certain chemical adsorption characteristics. In contrast, CO_2_ exhibits the weakest adsorption energy, measured at −0.170 eV, which primarily indicates physical adsorption. The adsorption energy of O_2_ is −1.553 eV, which is between NO_2_ and NO, suggesting that it has a stronger interaction than SO_2_, CO, and CO_2_, but a weaker interaction than NO_2_ and NO. The notable differences in adsorption energies endow CuO-SnS with excellent selectivity in gas sensing applications.

#### 3.2.2. Electronic Structure Analysis

**Figure 5:**
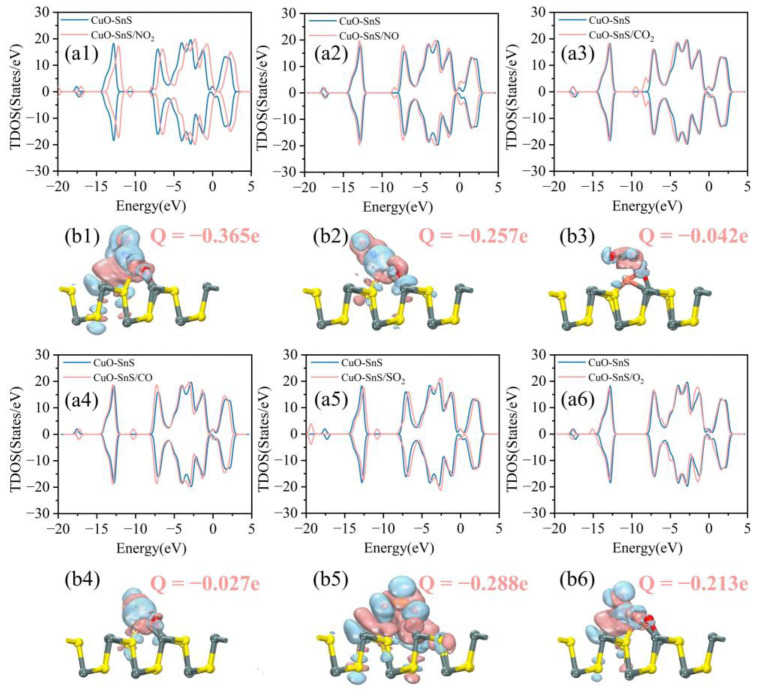
The TDOS of the system after gas adsorption by CuO-SnS: (**a1**) NO_2_ system, (**a2**) NO system, (**a3**) CO_2_ system, (**a4**) CO system, (**a5**) SO_2_ system, (**a6**) O_2_ system. The DCD of the system after gas adsorption by CuO-SnS: (**b1**) NO_2_ system, (**b2**) NO system, (**b3**) CO_2_ system, (**b4**) CO system, (**b5**) SO_2_ system, (**b6**) O_2_ system.

To investigate the electronic characteristics before and after adsorption, we conducted a detailed analysis of the TDOS and differential charge density (DCD) for the CuO-SnS monolayer adsorbed with five different gases. From Figure 5(a1,a5) showing the TDOS, it is observed that new peaks emerge near the Fermi level following the adsorption of NO_2_, indicating significant orbital hybridization during the adsorption processes, both in the spin-up and spin-down channels. This is further supported by the corresponding DCD in Figure 5(b1), which shows a large charge transfer of −0.365 e. These findings suggest a strong interaction between NO_2_ molecules and the CuO-SnS surface during adsorption. Similarly, following the adsorption of SO_2_, the TDOS of the system undergoes a shift to the right. The charge transfer amount (Q) is measured at −0.288 e, which points to a robust chemical adsorption of SO_2_ on the CuO-SnS surface. For NO and CO, as shown in Figure 5(b2,b4) showing the DCD, the blue and pink regions are relatively small, indicating a decreased adsorption strength compared to NO_2_ and SO_2_. The charge transfer amounts are −0.257 e and −0.027 e for CO_2_ and CO, respectively. Concerning the adsorption of CO_2_, there are minimal changes in the TDOS, with no notable emergence of new peaks in the vicinity of the Fermi level, whether in the spin-up or spin-down channels. The DCD (Figure 5(b3)) reveals a small amount of charge redistribution (*Q* = −0.042 e), indicating that the adsorption is mostly physical in nature. This means that the connection between the surfaces is weaker and relies on van der Waals forces. Additionally, the adsorption of O_2_ also reveals significant electronic characteristics. The TDOS after O_2_ adsorption exhibits spin asymmetry, indicating the presence of magnetic moments induced by the adsorbed O_2_ molecules. This spin asymmetry is a clear indication of the magnetic interaction between O_2_ and the CuO-SnS surface, as shown in Figure 5(a6).

**Figure 6:**
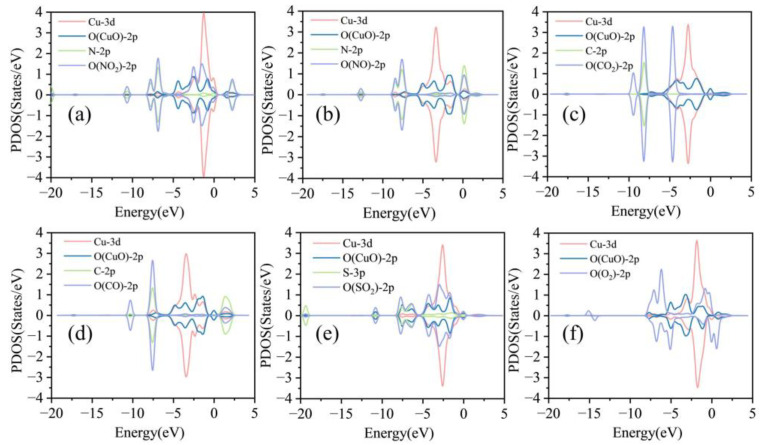
The PTDOS of the system after gas adsorption by CuO-SnS: (**a**) NO_2_ system, (**b**) NO system, (**c**) CO_2_ system, (**d**) CO system, (**e**) SO_2_ system, (**f**) O_2_ system.

Examining the PDOS of a CuO-SnS monolayer adsorbed with six different gases provides deeper insights into the interaction mechanisms [43,44]. It should be noted that NO_2_, NO, CO_2_, CO, and SO_2_ do not exhibit magnetism on the CuO-SnS surface, and therefore the spin-up and spin-down states are analyzed together. Following NO₂ adsorption, significant hybridization occurs between Cu-3d orbitals and O (NO_2_)-2p orbitals near −2 eV, resulting in the formation of new peaks, as shown in Figure 6a. This phenomenon indicates robust chemical adsorption between NO_2_ molecules and the CuO-SnS surface, leading to substantial electron rearrangement and pronounced modifications in the system’s electronic states. Similarly, SO_2_ adsorption, as shown in Figure 6e, exhibits pronounced hybridization between Cu-3d and O (CuO)-2p orbitals in the energy range of −5 eV to 0 eV, along with significant contributions from O (SO_2_)-2p and S-3p orbitals. This strong orbital interaction confirms the high adsorption strength of SO_2_ on the CuO-SnS surface. For NO adsorption, as shown in Figure 6b, hybridization between Cu-3d, N-2p, and O (NO)-2p orbitals near the Fermi level suggests moderate chemical adsorption. CO adsorption also exhibits moderate-strength interactions, though it is less pronounced than NO. In stark contrast, CO_2_ adsorption, as shown in Figure 6c, shows no apparent atomic orbital hybridization, aligning with its weak adsorption energy and minimal electronic interaction with the surface. O_2_ adsorption, as shown in Figure 6f, exhibits unique hybridization behavior compared to the other gases. In the spin-up region, O (O_2_)-2p orbitals hybridize with Cu-3d orbitals within the energy range of −2.5 eV to 0 eV, while in the spin-down region, hybridization occurs predominantly near the Fermi level. This spin-polarized interaction suggests a dual mechanism, with localized charge transfer in the spin-up channel and Fermi level coupling in the spin-down channel. However, the overall hybridization intensity is weaker than that of NO_2_ or SO_2_.

**Figure 7:**
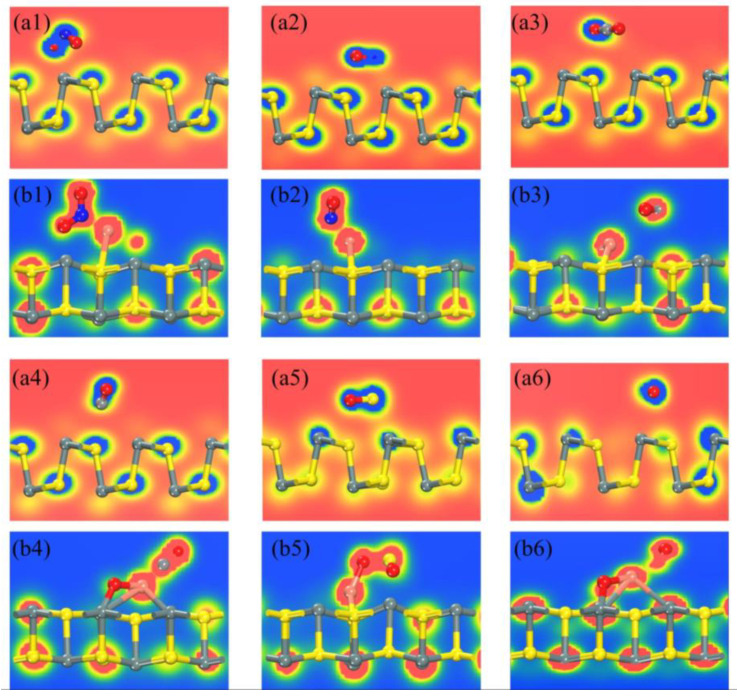
The ELF of the system after gas adsorption: (**a1**) NO_2_, (**a2**) NO, (**a3**) CO_2_, (**a4**) CO, (**a5**) SO_2_, and (**a6**) O_2_ adsorbed on pure SnS; (**b1**) NO_2_, (**b2**) NO, (**b3**) CO_2_, (**b4**) CO, (**b5**) SO_2_, and (**b6**) O_2_ adsorbed on CuO-SnS.

To further investigate the adsorption mechanism of gas molecules on both intrinsic SnS and CuO-modified SnS surfaces, we employed the electron localization function (ELF) to qualitatively characterize the electronic structure of gas adsorption (Figure 7). The ELF function ranges from 0 to 1, where ELF = 1 represents a fully localized electronic state, while ELF = 0 corresponds to a completely delocalized or zero electron density region. A comparative analysis of Figure 7(a1,a2) reveals that for the intrinsic SnS surface, none of the six gas molecules induce significant electron localization upon adsorption. This suggests a weak interfacial interaction, indicative of physisorption.

Notably, CuO surface modification significantly alters the adsorption characteristics of the system. Among the studied gases, SO_2_ exhibits a continuous high-ELF region in red, indicating strong chemical bonding with the SnS surface. Meanwhile, NO_2_ and NO display moderately strong electron localization, characteristic of chemisorption. As shown in Figure 7(b6), the ELF value for O_2_ on the CuO-SnS surface (ELF ≈ 0.35) is considerably lower than that of most other gas-substrate systems, except for CO_2_. This suggests that O_2_ undergoes only weak physisorption on the modified surface. These findings strongly confirm that the introduction of CuO effectively enhances the specific adsorption capacity of the SnS surface for target pollutants such as SO_2_ and NO_X_ by introducing new active sites.

#### 3.2.3. Conductivity Analysis

**Figure 8:**
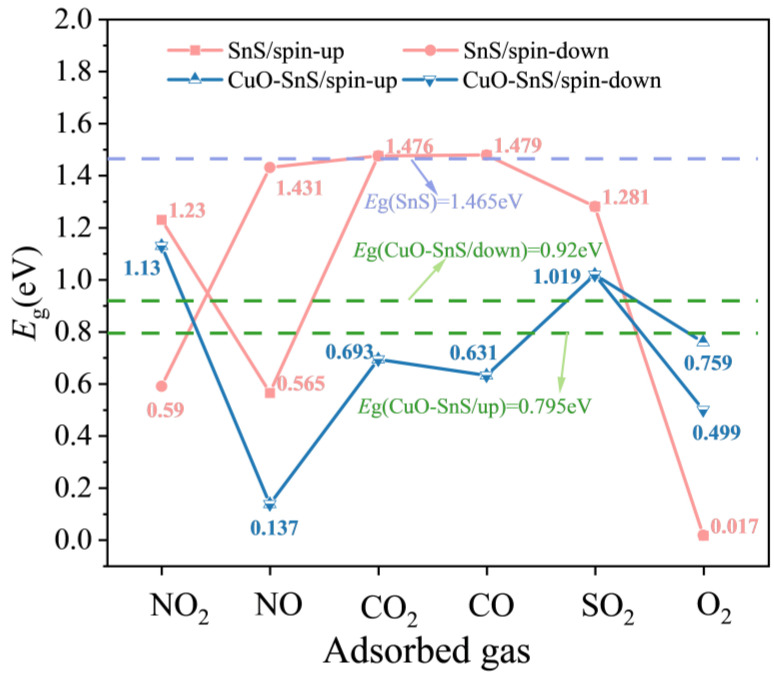
Band gap evolution and conductivity changes of pure SnS and CuO-SnS system before and after gas adsorption.

To elucidate the regulatory mechanism of CuO modification in the electronic transport properties of SnS, this study systematically analyzes the band gap variations before and after gas adsorption and their structure–conductivity relationships, as shown in Figure 8. In the pristine SnS system, the adsorption of NO and NO_2_ induces significant spin polarization, leading to band splitting into two spin channels. Among them, the spin-down channel exhibits the most pronounced band gap reduction, which is closely associated with the enhancement of its magnetic moment and the redistribution of charge.

Upon CuO modification, the system demonstrates more intricate band structure regulation. The introduction of CuO doping results in the splitting of the originally single band into two characteristic branches, with the spin-up channel exhibiting a band gap of 0.795 eV. In the cases of CO_2_ and CO adsorption, the band gap variation rates relative to the spin-up band of the substrate are 13.4% and 16.9%, respectively, indicating the weak surface interactions of these gases. In stark contrast, the NO adsorption system undergoes a dramatic band gap reduction of up to 81.9%. This phenomenon can be attributed to the formation of spin-selective transport channels at the CuO-SnS interface, providing additional pathways for electron transport and significantly enhancing the electrical conductivity of the system. However, in the O_2_ adsorption system, CuO modification exhibits a distinct trend in conductivity regulation. Compared to the pristine SnS system, the conductivity of CuO-modified SnS decreases drastically by several orders of magnitude. Theoretical calculations suggest that this phenomenon may be closely related to defect state capture effects induced by CuO and the redistribution of carrier concentration, further elucidating the complex modulation mechanism of gas adsorption and interfacial modification in material conductivity. These findings provide critical theoretical insights for the design of high-performance spintronic devices, particularly in the optimization of spin-selective transport and carrier mobility, highlighting their significant application potential.

### 3.3. Desorption Performance Analysis

(5) $$\tau ={v_{0}}^{-1}\exp(-E_{\mathrm{ads}}/KT)$$

where *τ* represents the recovery time, *V*₀ is the attempt frequency set to 10^12^ s⁻^1^, *K* is the Boltzmann constant (8.62 × 10^−5^ eV/K), and *T* denotes the temperature of the sensitive material [45]. The equation demonstrates that the desorption time shortens with rising temperature.

**Figure 9:**
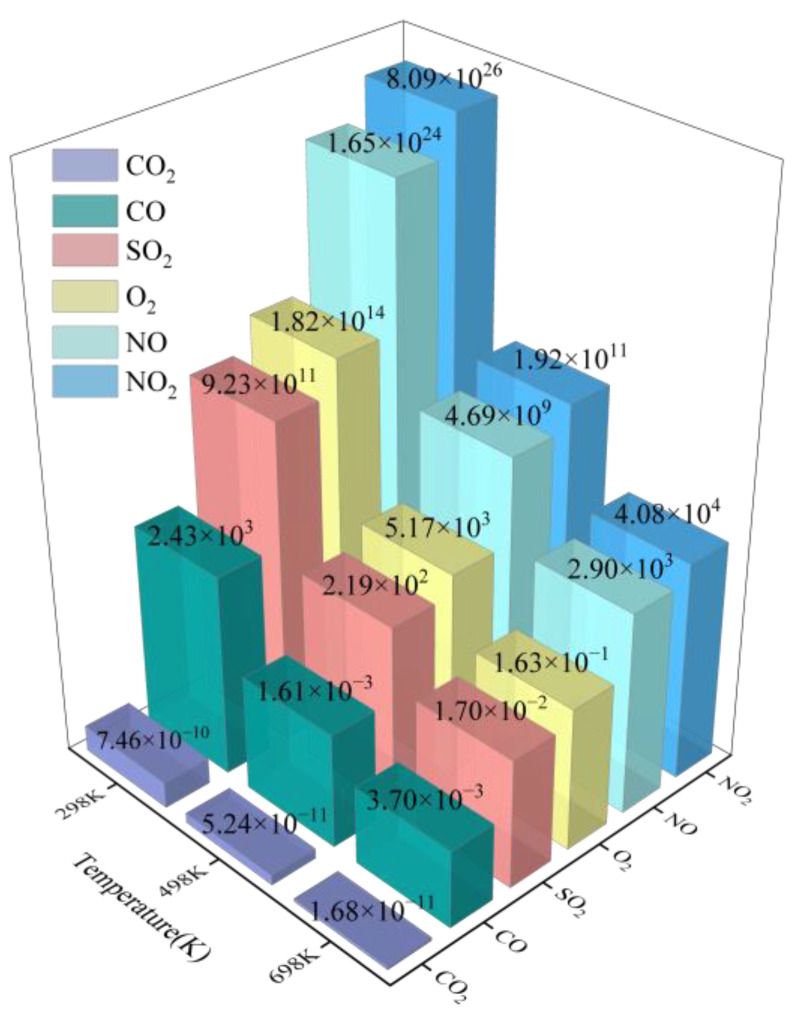
Recovery times of various adsorption systems on the CuO-SnS substrate.

Figure 9 visually depicts the desorption time of various gas molecules on CuO-SnS monolayers at different temperatures. At 698 K, the desorption time of NO_2_ dramatically decreases from 8.09 × 10^26^ s at 298 K to 4.08 × 10^4^ s. Similarly, the desorption time of NO reduces from 1.65 × 10^24^ s to 2.90 × 10^3^ s. O_2_ and SO_2_ also exhibit notable temperature-dependent desorption behavior. Meanwhile, it is evident that CO can achieve the most favorable desorption time at some temperature point within the range of 298 K and 498 K. These results demonstrate the recyclable nature of the adsorbent, but it requires recovery and reuse under high-temperature conditions. In contrast, the desorption time for CO_2_ on the CuO-SnS monolayer is very short at room temperature. At 298 K, the desorption time is only 7.46 × 10^−10^ s, indicating very weak adsorption of CO_2_ under ambient conditions, making it difficult to effectively capture CO_2_ molecules. In summary, CuO-SnS monolayers exhibit excellent high-temperature gas sensing performance with rapid response and recovery to target specific gases, demonstrating their potential as efficient and reusable gas sensor materials.

## 4. Conclusions

In this paper, the effects of gases (NO_2_, NO, CO_2_, CO, SO_2_, O_2_) on CuO-SnS monolayers were systematically investigated.

Based on an analysis of the band structures and density of states, the introduction of CuO notably reduces the band gap of SnS and increases the electronic density of states near the Fermi energy level, which enhances the conductivity. Comparison results reveal that CuO-modified SnS exhibits significantly better adsorption performance for target toxic gases than pure SnS.

The analyses of TDOS, PDOS, and DCD before and after adsorption reveal that the adsorption of NO_2_ and SO_2_ molecules results in substantial charge transfer and orbital hybridization. This offers information on the intrinsic mechanism of the interaction between the gas molecules and the CuO-SnS monolayers, providing a theoretical basis for CuO-SnS monolayers used in gas detection.

The adsorption properties of the CuO-SnS monolayers vary significantly for different gases, showing the highest sensitivity for the hazardous gases NO_2_ and SO_2_, and the lowest sensitivity for CO_2_. Moreover, in competition with O_2_, NO_2_ and NO remain dominant. These findings render CuO-SnS monolayers a potential candidate for NO_X_ gas sensors.

## Figures and Tables

**Table 1:** Adsorption property parameters.

System	Structure	d(Å)	*E*_ads_(eV)
CuO-SnS/NO_2_	N-Cu	N-Cu:1.904	−2.301
CuO-SnS/NO	N-Cu	N-Cu:1.841	−2.142
CuO-SnS/CO_2_	C-Cu	C-Cu:3.476	−0.170
CuO-SnS/CO	C-Cu	C-Cu:1.832	−0.910
CuO-SnS/SO_2_	S-Cu	S-Cu:2.919	−1.417
CuO-SnS/O_2_	O-O	O-O:4.617	−1.553

## Data Availability

Data will be made available on request.

## Supplementary Materials

The following supporting information can be downloaded at https://www.mdpi.com/article/10.3390/s25051439/s1: Table S1: The *E*_b_ of various SnS-CuO monolayer structures. Table S2: The *E*_ads_ of gases on SnS. Table S3: The *E*_ads_ of gases on CuO-SnS.
