# Supplementary material for: Adsorption and Detection of Toxic Gases on CuO-Modified SnS Monolayers: A DFT Study
Source: Sensors (Basel). 2025 Feb 26;25(5):1439. doi: 10.3390/s25051439 (PMC11902759; doi:10.3390/s25051439)
Supplement: Supplementary file 1 [file sensors-25-01439-s001.zip › sensors-3440838-supplementary.pdf]

sensors

MDPI

Supplementary Materials

Adsorption and Detection of Toxic Gases on CuO-Modified SnS Monolayers: A DFT Study

Xinyue Liang [1], Ping Wang [1,*], Kai Zheng [1,*], Xuan Yang [2], Meidan Luo [2], Jiaying Wang [2], Yujuan He [2], Jiabing Yu [2] and Xianping Chen [1,2]

[1] The State Key Laboratory of Power Transmission Equipment & System Security and New Technology, Chongqing University, Chongqing 400044, China; xianpingchen@cqu.edu.cn (X.C.)

[2] Key Laboratory of Optoelectronic Technology & Systems, Education Ministry of China, College of Optoelectronic Engineering, , Chongqing University, Chongqing 400044, China; yujiab@cqu.edu.cn (J.Y.)

* Correspondence: cqu_dqwp@163.com (P.W.); kzhengmailbox@163.com (K.Z.)

To identify the optimal binding sites for CuO-modified surfaces, we constructed models of various binding sites to encompass all possible models, positioning the Cu and O atoms above the S and O atoms of the substrate, respectively. The most stable structure was ultimately determined by the maximum absolute value of the binding energy, with larger absolute values indicating more stable systems, as calculated using the formula provided in Equation (1). By comparing the binding energies of all models, as shown in Table S1, we were able to identify the optimal modification sites. The energy released (E_b) during the binding process of CuO with the SnS surface is defined by following equation:

$$E_b = E_{CuO\text{-}SnS} - E_{SnS} - E_{CuO} \quad (S1)$$

where $E_{CuO\text{-}SnS}$, E_{SnS}, and E_{CuO} represent the CuO-SnS monolayer, pure SnS monolayer, and CuO unit, respectively. When E_b is negative, it indicates that the binding process between CuO and SnS is accompanied by a net energy release, and the system spontaneously evolves toward a stable state, following the second law of thermodynamics. The larger the absolute value of E_b, the stronger the chemical bonding interaction at the CuO-SnS interface, leading to a more stable structure of the composite system.

Table S1. The E_b of various SnS-CuO monolayer structures.

Scheme	Structure	E_b(eV)
SnS-CuO	Cu-S	-3.572
	Cu-Sn	-3.426
	O-S	-3.532
	O-Sn	-3.396

To determine the optimal adsorption sites for gas molecules, we systematically constructed models representing various adsorption configurations. Taking NO_2 adsorption on SnS as a representative case, we positioned the N and O atoms of the gas molecule above the S and O atoms of the substrate, respectively, and applied a similar approach for other gases. Based on Definition (2), adsorption sites with higher absolute values of adsorption energy were preliminarily identified as exhibiting superior adsorption performance. Ultimately, the most favorable adsorption site was determined by the maximum absolute value of adsorption energy. The adsorption energies for each adsorption model on SnS and CuO-SnS surfaces are summarized in Tables S2 and S3, respectively.

The adsorption energy (E_{ads}) is calculated using definition (2):

$$E_{\text{ads}} = E_{\text{Total}} - E_{\text{Sub}} - E_{\text{Gas}} \tag{S2}$$

where E_{Total}, E_{Sub}, and E_{Gas} represent the energy of the doped system following gas adsorption, the energy of the standalone substrate system, and the energy of the individual gas, respectively. If E_{ads} is negative, it indicates energy release during the reaction process, making the process spontaneous.

Table S2. The E_{ads} of gases on SnS.

System	Structure	E_{ads}(eV)
SnS/NO_2	N-S	-1.134
	N-Sn	-0.893
	O-S	-1.135
	O-Sn	-0.737
SnS/NO	N-S	-0.441
	N-Sn	-0.519
	O-S	-0.523
	O-Sn	-0.437
SnS/CO_2	C-S	-0.216
	C-Sn	-0.136
	O-S	-0.190
	O-Sn	-0.171
SnS/CO	C-S	-0.231
	C-Sn	-0.233
	O-S	-0.167
	O-Sn	-0.115
SnS/SO_2	S-S	-0.487
	S-Sn	-0.577
	O-S	-0.577
	O-Sn	-0.485
SnS/O_2	O-S	-0.624
	O-Sn	-0.689

Table S3. The E_{ads} of gases on CuO-SnS.

System	Structure	E_{ads}(eV)
SnS-CUO/NO_2	N-Cu	-2.301
	N-O	-1.993
	O-O	-2.172
	O-Cu	-1.964
SnS-CUO/NO	N-Cu	-2.142
	N-O	-2.074
	O-Cu	-1.061
	O-O	-1.929
SnS-CUO/CO2	C-Cu	-0.170
	C-O	-0.158
	O-Cu	-0.053
	O-O	-0.056
SnS-CUO/CO	C-Cu	-0.910
	O-Cu	-0.052
	C-O	0.039

	O-O	0.102
SnS-CUO/SO2	S-Cu	-1.417
	S-O	-0.529
	O-Cu	-1.415
	O-O	-1.401
SnS-CUO/O2	Cu-O	-1.401
	O-O	-1.553
